# Application of the Machine-Learning Model to Improve Prediction of Non-Sentinel Lymph Node Metastasis Status Among Breast Cancer Patients

**DOI:** 10.3389/fsurg.2022.797377

**Published:** 2022-04-25

**Authors:** Qian Wu, Li Deng, Ying Jiang, Hongwei Zhang

**Affiliations:** ^1^Department of General Surgery, Shanghai Public Health Center, Shanghai, China; ^2^Department of General Surgery, Zhongshan Hospital, Fudan University, Shanghai, China

**Keywords:** breast neoplasms, sentinel lymph node, nomogram, ultrasound, machine learning

## Abstract

**Background:**

Performing axillary lymph node dissection (ALND) is the current standard option after a positive sentinel lymph node (SLN). However, whether 1–2 metastatic SLNs require ALND is debatable. The probability of metastasis in non-sentinel lymph nodes (NSLNs) can be calculated using nomograms. In this study, we developed an individualized model using machine-learning (ML) methods to select potential variables, which influence NSLN metastasis.

**Materials and Methods:**

Cohorts of patients with early breast cancer who underwent SLN biopsy and ALND between 2012 and 2021 were created (training cohort, N 157 and validation cohort, N 58) for the development of the nomogram. Three ML methods were trained in the training set to create a strong predictive model. Finally, the multiple iterations of the least absolute shrinkage and selection operator regression method were used to determine the variables associated with NSLN status.

**Results:**

Four independent variables (positive SLN number, absence of lymph node hilum, lymphovascular invasion (LVI), and total number of SLNs harvested) were combined to generate the nomogram. The area under the receiver operating characteristic curve (AUC) value of 0.759 was obtained in the entire set. The AUC values for the training set and the test set were 0.782 and 0.705, respectively. The Hosmer-Lemeshow test of the model fit accuracy was identified with *p* = 0.759.

**Conclusion:**

This study developed a nomogram that incorporates ultrasound (US)-related variables using the ML method and serves to clinically predict the non-metastatic status of NSLN and help in the selection of the appropriate treatment option.

## Introduction

In 2020, breast cancer was surpassed lung cancer as the world's most commonly diagnosed cancer. This is despite being arguably negligible in men (1). The most common route of breast cancer metastasis is lymphatic spread within the axilla. Axillary lymph node dissection (ALND) and sentinel lymph node biopsy (SLNB) are the main axillary surgeries for breast cancer (2). ALND could completely remove the metastatic lymph nodes, clarify the TNM stage of the cancer, and inform the prognosis (3). However, ALND can cause many complications, such as lymphedema, hematoma, sensory abnormalities, and limitation of upper limb movement (4, 5). Furthermore, approximately 50% of patients with positive sentinel lymph nodes (SLNs) are found to have no additional nodal metastases (6).

The possibility of exempting ALND in early breast cancer (cT1-2N0) has been widely explored in several clinical trials (7–10). This suggestion has essentially achieved good follow-up data reports. According to St. Gallen guidelines of 2017, women with 1 or 2 positive SLNs who have had breast conservation can avoid ALND and receive whole breast radiation and adjuvant systemic therapies only (11). However, with the limited randomized, multicenter clinical trials and strict inclusion criteria, proper selection of axillary surgeries for patients who fail to meet the criteria has become a priority of many clinicians.

The prediction of the risk of non-sentinel lymph nodes (NSLNs) metastasis determines the selection of axillary surgery. Previous reports show that nomograms have been developed and are validated as the commonly used method of predicting cancer prognosis. The Memorial Sloan-Kettering Cancer Center (MSKCC) nomogram (12) is undoubtedly the most authoritative nomogram. Different cancer centers have validated and confirmed MSKCC nomogram as a robust method of predicting NSLNs metastasis (13, 14).

Multivariate logistic regression is the most common method of incorporating variables into cancer prognostication. Machine learning (ML) is an emerging tool for predicting cancer prognosis that is making significant contributions in different cancer fields (15, 16). It is a learning process, which utilized techniques, such as decision trees (DTs), artificial neural networks (ANNs), and support vector machines (SVMs). Ayer et al. applied the ANN technique in the prediction of breast cancer susceptibility (17) while Zeng et al. predicted the breast cancer recurrence through SVMs (18). Further, Madekivi et al. filtered the variables by a gradient-boosted trees model to develop a final model for predicting NSLNs (19). All these studies show ML as a feasible and superior cancer prediction method. The aim of our study was to employ ML-based statistical methods to select variables with potential influence on NSLN metastasis status. The study ultimately developed an individualized prediction model that could guide clinicians for a better choice of cancer treatment options for different patients.

## Method

### Patients and Data Collection

The clinical data of patients who underwent surgery between January 2012 and May 2021 at Zhongshan Hospital (an affiliate of Fudan University) and Shanghai Public Health Center (Zhongshan Hospital South Branch) were collected and retrospectively analyzed. A total of 532 patients were screened (*n* = 532). The inclusion criteria were postoperative pathologically confirmed diagnosis of primary breast cancer, no history of other tumors, and that the patient has received both SLNB and ALND.

**Figure 1:**
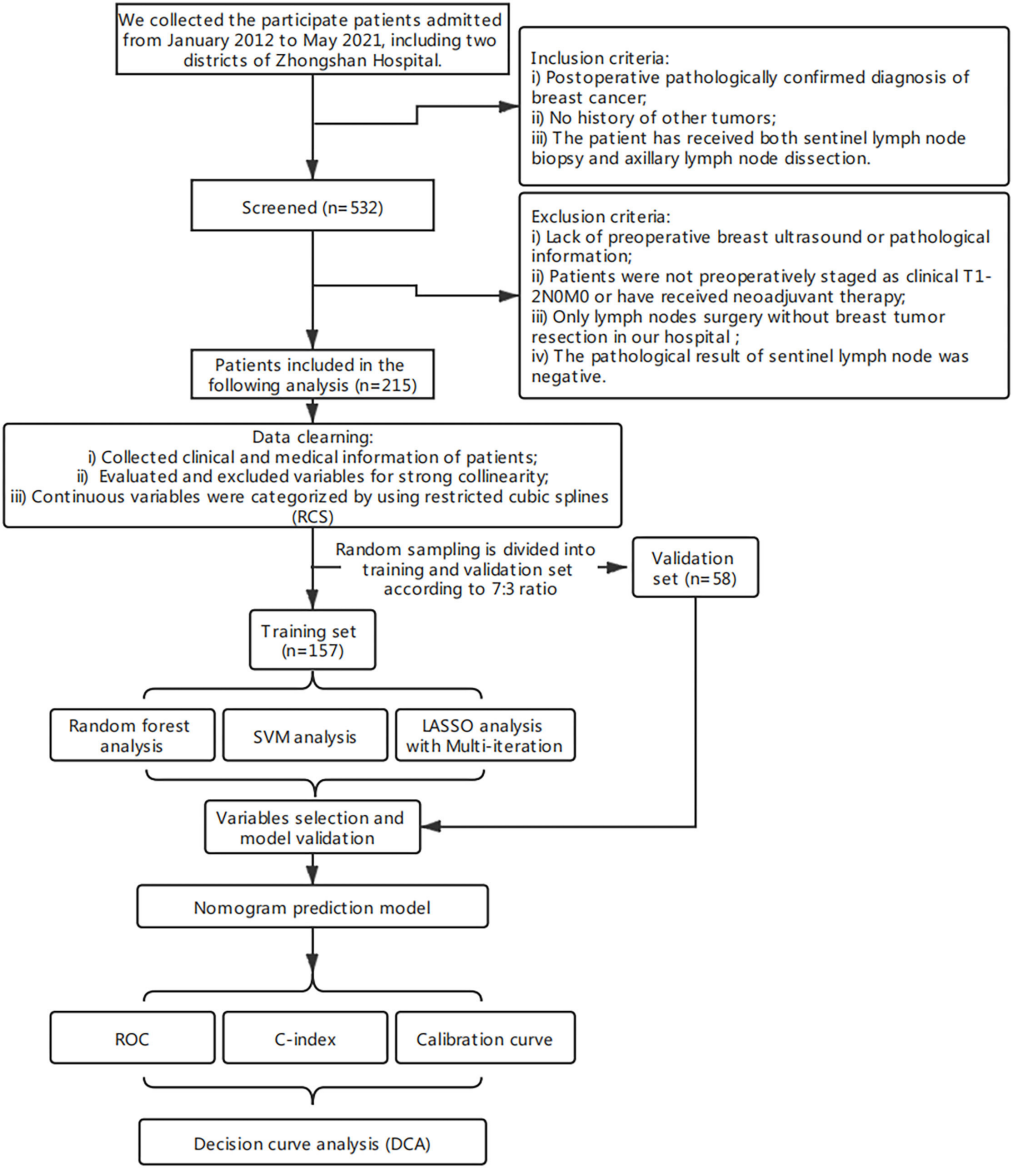
The working flow of this study.

The exclusion criteria were lack of preoperative breast ultrasound (US) or pathological information, patients were not preoperatively staging as clinical T1-2N0 or had received neoadjuvant therapy, only axillary surgery without breast tumor resection in our hospital, and a negative pathological result of SLNs. Only 215 patients were retained by the exclusion criteria. The working protocol of our study is as shown in Figure 1. The ethical approval of this study was granted by the ethics committee of Zhongshan Hospital and Shanghai Public Health Center. There was no additional informed consent required from the patients because this was a retrospective study.

The SLNs were identified before surgery using Methylene blue dye, and blue-stained nodes were removed and sent for frozen pathology to the Pathology Department. It was stained with H&E and microscopically examined by an experienced pathologist. Routine H&E analysis was performed for all additional nodes identified by ALND.

The collected clinical and medical information of patients included the patient age, breast tumor location, postoperative pathological features [histology, estrogen receptor (ER) status, proliferation index (Ki-67), progesterone receptor (PR) status, human epidermal growth factor receptor 2 (HER-2) overexpression, lymphovascular invasion (LVI), Scarff-Bloom-Richardson (SBR) grade, total number of SLNs harvested, T stage, number of positive SLNs, and number of NSLNs metastasis] and ultrasonic parameters of tumor and axillary lymph nodes [sizes, mass echogenicity, regular or irregular tumor margin, presence or absence of lymph node hilum, and color Doppler flow imaging (CDFI)].

### Statistical Analysis

Clinical and pathological variables associated with the risk of lymph node metastasis were assessed on the basis of their clinical importance and predictors identified in previously published articles (20, 21). Categorical variables were reported as integers and proportions. The continuous variables were described as means [±standard deviation (SD)]. Collinearity for all explanatory variables was assessed using a correlation matrix and plausible interaction terms were also tested. Therefore, interaction terms were excluded in the multivariate analysis. To relax the assumption of a linear relationship between continuous predictors and the risk of NSLN metastasis status, continuous predictors that include the patient age, tumor size, and number of SLNs, etc., were categorized after evaluation using restricted cubic splines (22). Regarding the strong U- or S-shaped relation between continuous predictors and NSLNs metastasis results, the value of the turning in the graph was used as the dividing point (Supplementary Figure S1).

Patients were randomly sampled into the training and validation sets in the ratio of 7:3. To select the strongest predictive model, three ML methods were trained in the training set. These ML methods were random forest (RF) (23), SVMs (24), multiple iterations of the least absolute shrinkage, and least absolute shrinkage and selection operator (LASSO) regression (25). The best hyper-parameter for ML models was 10-fold cross-validation to avoid overfitting. The best classification model was selected to compare the performance of the ML methods.

We created a nomogram that could make a linear predictor in patients who were easily accessible to clinicians on the basis of the best-performing model. Further, we assessed the discriminating ability and predictive accuracy in both the validation and entire sets using the ROC curves (C-index and calibration curves) (26, 27). Finally, the decision curve analysis (DCA) was used to support the clinical decisions of the prepared prediction model (28). All the statistical analyses were carried out using the R software (version 3.6.3, http://www.r-project.org). The R-software packages used for statistical data analysis were “caret,” “rms,” “glmnet,” “randomForest,” “e1071,” “kernlab,” “pROC,” “rmda,” and “ResourceSelection.” A two-sided *p* < 0.05 was considered statistically significant.

## Results

### Demographic and Clinical Characteristics

Clinicopathological information of 215 patients with breast cancer after surgical operation was assessed between January 2012 and May 2021. Patients who participated were grouped into two groups based on the presence or absence of NSLNs metastasis. The observed clinicopathological characteristics of both metastasis and non-metastasis patients are presented in Table 1. All patients are Asian. The median age of patient was 56 years. The patients with tumors located in the upper-outer quadrant were 51.2%. Largely, all participating patients (97.6%) were at the T1-T2 stages and showed no difference in histology, ER, PR, and ki-67 or HER-2 status. A small proportion of patients in stage T3 were included as the preoperative assessment was T1-T2 but the postoperative pathology confirmed stage T3. However, the ultrasonic features (longitudinal diameter of lymph node, lymphatic echogenicity, and absence of lymph node hilum) and pathological features (LVI, number of positive SLNs, and proportion of positive SLNs) in the two groups were statistically different (*p* < 0.05).

**Table 1:** Differences in clinicopathological characteristics between the patients with and without NSLNs metastasis.

	**Metastasis (*n =* 96)**	**Non-Metastasis (*n =* 119)**	**all (*n =* 215)**	***P*-value**
Age	56.3 (12.2)	55.7 (11.7)	56 (11.9)	0.703
**Tumor location**
UOQ	51 (53.1%)	59 (49.6%)	110 (51.2%)	
LOQ	18 (18.8%)	25 (21.0%)	43 (20.0%)	
UIQ	15 (15.6%)	25 (21.0%)	40 (18.6%)	
LIQ	12 (12.5%)	10 (8.4%)	22 (10.2%)	0.58
**Ultrasonic features**
Transverse diameter of tumor (mm)	23.5 (10.3)	21.5 (9.1)	22.4 (9.7)	0.115
Longitudinal diameter of tumor (mm)	14.6 (5.8)	14.4 (5.9)	14.5 (5.9)	0.672
longitudinal/transverse axis ratio of tumor	1.7 (0.5)	1.5 (0.5)	1.6 (0.5)	0.052
**Tumor margin**
regular	8 (8.3%)	7 (5.9%)	15 (7.0%)	
irregular	88 (91.7%)	112 (94.1%)	200 (93.0%)	0.666
Tumor CDFI	0.8 (0.1)	0.8 (0.1)	0.8 (0.1)	0.593
Transverse diameter of lymph nodes (mm)	11 (7.1)	9.5 (7.1)	10.2 (7.1)	0.16
Longitudinal diameter of lymph nodes (mm)	6.1 (4)	4.9 (3.8)	5.5 (4)	0.024
Longitudinal/transverse axis ratio of lymph nodes	1.6 (0.9)	1.5 (1)	1.5 (1)	0.923
**Lymphatic echogenicity**
None	17 (17.7%)	32 (26.9%)	49 (22.8%)	
High	18 (18.8%)	37 (31.1%)	55 (25.6%)	
Low	59 (61.5%)	49 (41.2%)	108 (50.2%)	
Moderate	2 (2.1%)	1 (0.8%)	3 (1.4%)	0.018
**Absence of lymph node hilum**
No or not described	82 (85.4%)	113 (95.0%)	195 (90.7%)	
Yes	14 (14.6%)	6 (5.0%)	20 (9.3%)	0.031
**Pathological features**
**Histology**
Ductal	92 (95.8%)	116 (97.5%)	208 (96.7%)	
Lobular	3 (3.1%)	3 (2.5%)	6 (2.8%)	
Others	1 (1.1%)	0 (0%)	1 (0.5%)	0.516
**Estrogen receptor status**
Negative	16 (16.7%)	21 (17.6%)	37 (17.2%)	
Positive	80 (83.3%)	98 (82.4%)	178 (82.8%)	0.994
**Progesterone receptor status**
Negative	31 (32.3%)	32 (26.9%)	63 (29.3%)	
Positive	65 (67.7%)	87 (73.1%)	152 (70.7%)	0.475
**Proliferation index (Ki-67)**
<14%	19 (19.8%)	24 (20.2%)	43 (20.0%)	
≥14%	77 (80.2%)	95 (79.8%)	172 (80.0%)	1
**Her-2 overexpression**
Negative	71 (74.0%)	90 (75.6%)	161 (74.9%)	
Positive	25 (26.0%)	29 (24.4%)	54 (25.1%)	0.902
**Lymphovascular invasion**
No	61 (63.5%)	100 (84.0%)	161 (74.9%)	
Yes	35 (36.5%)	19 (16.0%)	54 (25.1%)	0.001
**SBR stage**
I	2 (2.1%)	1 (0.8%)	3 (1.4%)	
II	48 (50.0%)	72 (60.5%)	120 (55.8%)	
III	46 (47.9%)	46 (38.7%)	92 (42.8%)	0.259
**T stage**
≤ 2 cm	39 (40.6%)	63 (52.9%)	102 (47.4%)	
2–5 cm	54 (56.2%)	54 (45.4%)	108 (50.2%)	
≥5 cm	3 (3.1%)	2 (1.7%)	5 (2.3%)	0.18
Number of SLNs harvested	5 (2.8)	5.4 (3.6)	5.2 (3.3)	0.728
Number of positive SLNs	2.7 (1.8)	1.7 (1.1)	2.1 (1.5)	0
Proportion of positive SLNs	0.6 (0.3)	0.4 (0.3)	0.5 (0.3)	0

*UOQ, Upper-outer quadrant; LOQ, Lower-outer quadrant; UIQ, Upper-inner quadrant; LIQ, Lower-inner quadrant; CDFI, color Doppler flow imaging; SBR grade, Scarff-Bloom-Richardson grade; SLNs, sentinel lymph nodes*.

### Predictive Model and Factors Selection

**Figure 2:**
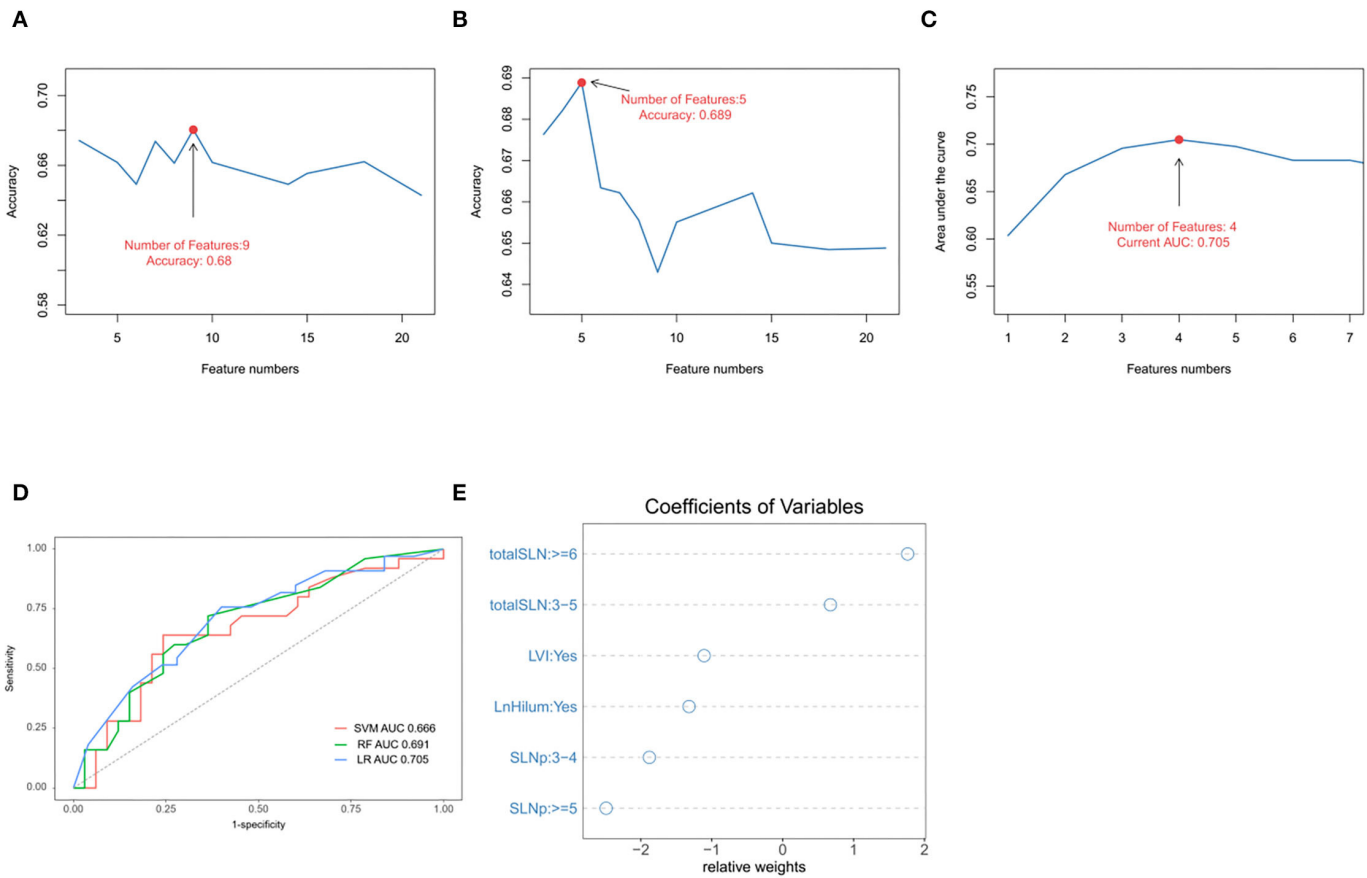
Predictive model and factors selection. **(A)** The line graph shows the relationship between the number of candidate features and the accuracy in support vector machines (SVMs) model. **(B)** The line graph shows the relationship between the number of selected features and the accuracy in random forest (RF) model. **(C)** The line graph shows the relationship between the number of features and the area under the curve (AUC) values in the least absolute shrinkage and selection operator (LASSO)-based logistical model. **(D)** ROC curve analysis of machine-learning algorithms for prediction of non-sentinel lymph nodes (NSLN) without metastasis in the validation set. **(E)** The dot plot shows the coefficients of variables in the final model. LVI: lymphovascular invasion; totalSLN: total number of SLNs harvested; LnHilum: absence of lymph node hilum; SLNp: number of positive SLNs.

All participants were randomly divided into two groups (training and validation cohorts) in the ratio of 7:3. The explanatory variables were transformed into categorical forms. The US transverse and longitudinal diameter of tumor or lymph were highly correlated, so only the largest diameter was retained. No other significant interaction was found. There were no statistical differences between the variables in both the training and validation sets (*p* > 0.05; Supplementary Table S1). Three ML algorithms were performed in the training set. The best SVM model was obtained when nine candidate variables were selected, as shown in Figure 2A. RF was effective in feature selection and the removal of redundant features. The RF model obtained the highest accuracy (0.689) with five predictive features (Figure 2B). The LASSO could select significantly predictive features but the results may not be identical each time. In this study, we conducted 500 times iterations and selected the features with more than 300 repeated occurrences. Then, these features were sequentially introduced into the logistic regression model to calculate the AUC values. The results showed that the final model with four predictive factors had the highest AUC of 0.705 in the validation set (Figure 2C). Comparisons of the predictive performance of validation sets among the three algorithms models (with each optimal variable and tuning parameter) are shown in Figure 2D and Supplementary Table S2. It turned out that the LASSO regression model demonstrated the highest performance. In detail, the relative weights of the final variables in the LASSO-based logistic regression model are displayed in Figure 2E.

### Nomograms and Model Performance

**Figure 3:**
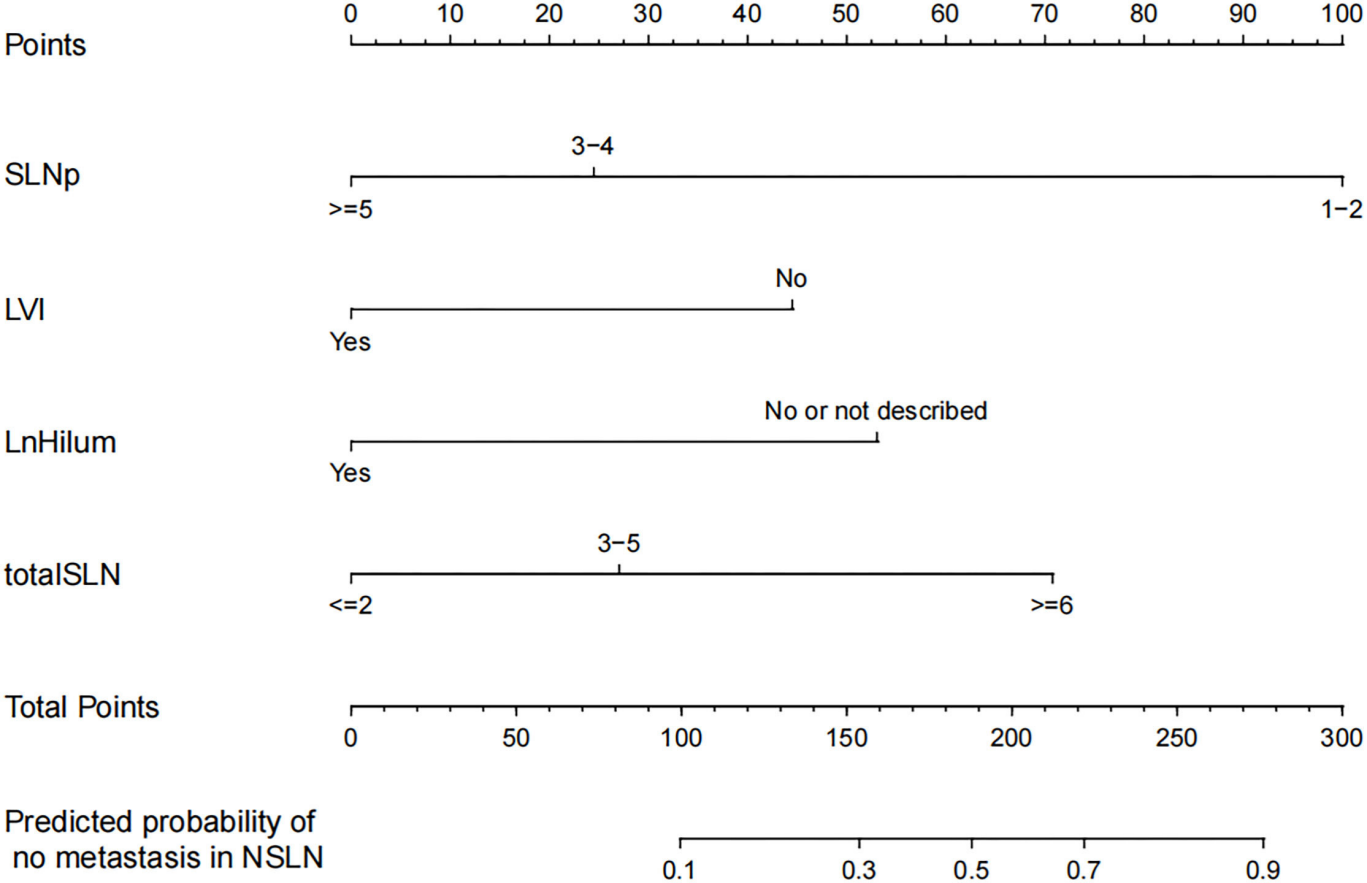
Nomogram for prediction of the absence of non-sentinel lymph nodes (NSLN) metastasis. SLNp: number of positive SLNs; LVI: lymphovascular invasion; LnHilum: absence of lymph node hilum; totalSLN: total number of SLNs harvested.

**Figure 4:**
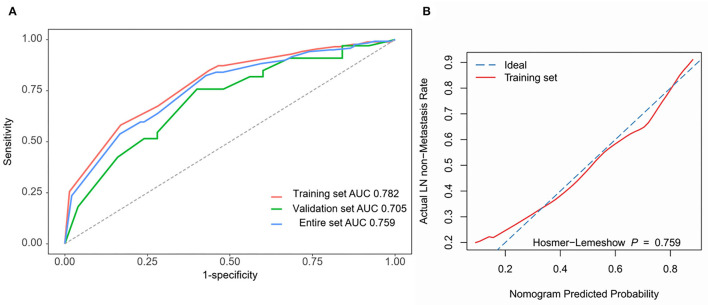
Nomogram prediction model validation. **(A)** ROC curves are used for all sets. AUC values for training set (red), validation set (green), and entire set (blue) are 0.782, 0.705, and 0.759. **(B)** The bootstrapped calibration plot and Hosmer-Lemeshow test for the training set.

The four independent factors used to create a predictive nomogram were the number of positive SLNs (1–2, 3–4, or ≥5), the total number of SLNs harvested (≤2, 3–5, or ≥6), absence of lymph node hilum (no/not described or yes), and LVI (no or yes). According to the sum of the assigned points for each factor in the nomogram, a higher total score was associated with the absence of NSLN metastasis (Figure 3). The c-index in the logistic regression was equal to the area under the ROC curve. In Figure 4A, an AUC value of 0.759 is achieved in the entire set, while AUC values of 0.782 and 0.705 are obtained in the training and validation sets, respectively. The Hosmer-Lemeshow test was used to assess the accuracy of model fit and no departure from perfect fit was identified (*p* = 0.759). The sample bootstrapped calibration plot for the prediction is also presented in Figure 4B.

### Clinical Application Evaluation

**Figure 5:**
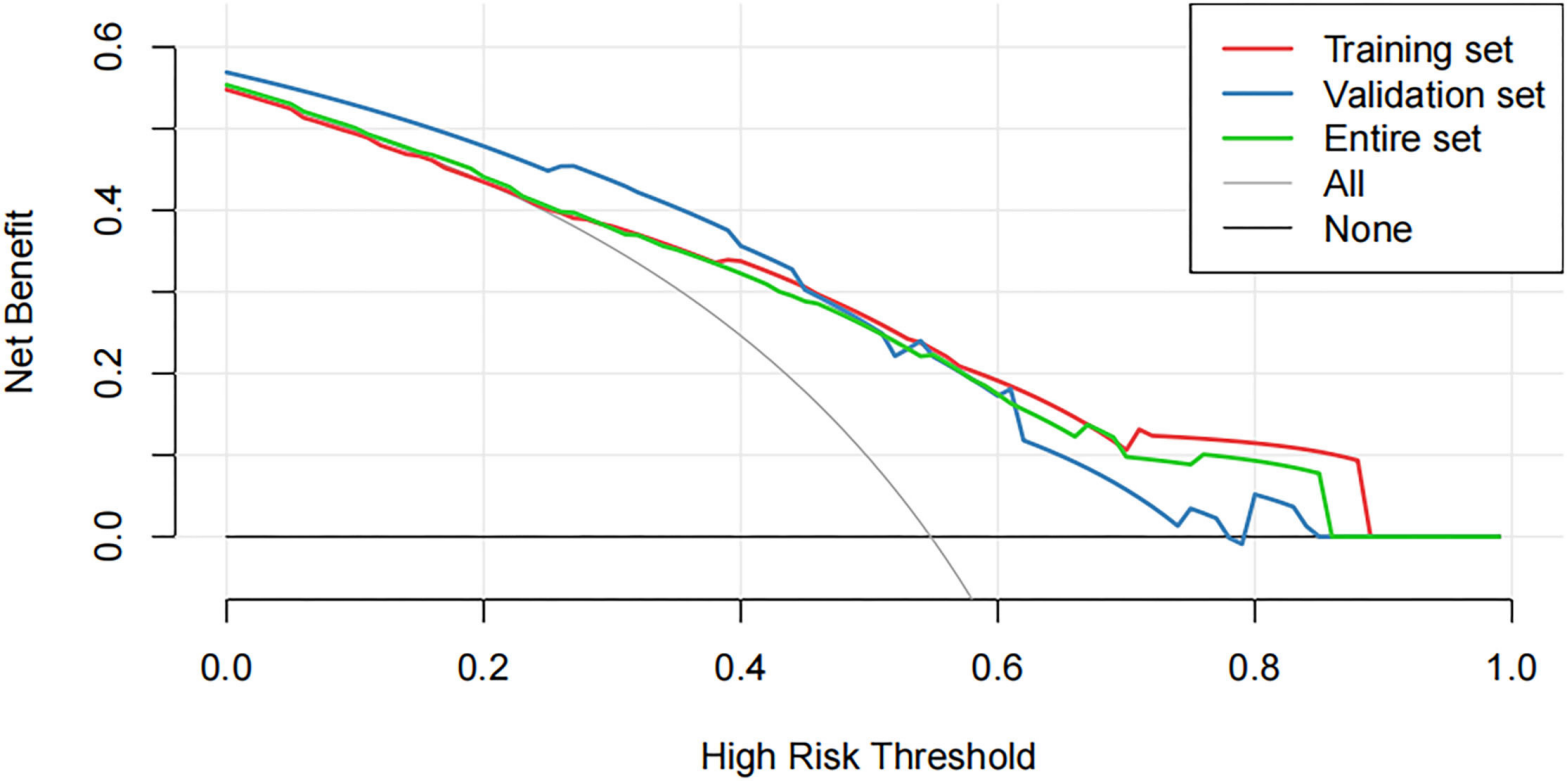
The decision curve analysis (DCA) curve shows the decision analysis for the entire set, the training set, and the validation set.

Decision curve analysis showed that using this nomogram provides an additional benefit when the threshold probability of the entire set is between 0 and 87% (Figure 5). A similar observation was also reported in entire and test cohorts. Therefore, the nomogram model can predict the probability of NSLN metastasis in patients with breast cancer to facilitate early clinical intervention and support personalized postoperative cancer rehabilitation.

## Discussion

The axillary surgery has been de-escalating since the awareness grows in breast cancer (29). Systemic treatments, such as chemotherapy therapies, are recognized as important control measures of cancer recurrence rather than the local therapy, such as the extent of surgical excision. The National Surgical Adjuvant Breast and Bowel Project (NSABP) b-32 trial (7) first investigated the necessity of ALND in patients with negative SLN. This trial found that SLNB alone without further axillary dissection is an appropriate therapy for the targeted patients. This report has been widely accepted in clinical practice to the point where it has become a surgical routine. Nevertheless, breast surgeons still explore the surgical indications. The After Mapping of the Axilla, Radiotherapy or Surgery (AMAROS) trial (8) revealed that ALND and axillary radiotherapy after a positive SLN provide comparable axillary control for patients with early breast cancer. The American College of Surgeons Oncology Group (ACOSOG) Z0011 (9) suggested that early breast cancer patients with 1 or 2 SLN metastases, breast-conserving lumpectomy, and whole-breast irradiation can be exempted from ALND. Furthermore, International Breast Cancer Study Group (IBCSG) 23-01 (10) trial argued the necessity of ALND in patients with micrometastatic SLN (metastases <2 mm). The discussion for sparing ALND is rooted in resultant series of complications that include range of motion, lymphoedema, pain, and sensory defects (4, 5, 30). In contrast, SLNB can significantly lower the morbidity of such complications (31). Furthermore, clinical data indicated that the majority of patients with positive SLNs had no additional nodes metastasis (6), which is consistent with our finding that roughly 55% of all patients had no metastasis. Despite the various clinical trials that explored the necessity of ALND, it is still apparent that the inclusion of patients is relatively stringent and the precise individualization of the choice is still pondering.

Previous studies have been conducted to predict NSLN metastasis (12, 20, 32–36). The MSKCC model is a widely acknowledged tool that incorporates eight variables (12). The variables combined in the MSKCC model were pathological size, ER status, multifocality, tumor type, tumor nuclear grade, LVI, method of detection, and the number of positive and negative SLNs with an AUC of 0.77 for the validation cohort. That the number of positive SLNs in the MSKCC model had the highest weight that is consistent with the findings of our study. The MSKCC model has been validated in various countries, for instance, in Australia where an AUC of 0.66 was obtained from the inclusion of 526 patients (14). The model of MD Anderson Cancer Center (33) is another frequently mentioned model. This model added two variables of SLN metastasis size and extracapsular extension, which are tied with the emphasis on SLN micrometastasis status. These variables were excluded in this study because some patients did not have SLN metastatic size as a result of the limitations of our pathology department. In contrast, the Helsinki University model (20) included a prediction variable of HER-2 status instead of PR status. Despite the fact that HER-2 positive is generally associated with NSLN positivity, the relationship between HER-2 status and NSLN positivity remains controversial (37), and the current study yielded no statistically significant differences.

Preoperative assessment of axillary lymph burden in breast cancer is routinely performed using different imaging techniques. US is considered to be the most recommended imaging technique owing to its inexpensive, convenience, and absence of radiation exposure. The Sentinel Node vs. Observation after Axillary Ultrasound (SOUND) trial (38) is exploring the potential possibility of US as a replacement for SLNB. Previous studies have also suggested that the inclusion of US parameters in the model could improve its predictive capacity (39, 40). Zhu (40) suggested that the Doppler resistance index and the extent of extension of the enhancing lesion were correlated with lymph node metastasis. Qiu et al. (39), on the other hand, incorporated three US-based variables of cortical thickness of SLN, transverse diameter of SLN, and lymph node hilum status in their nomogram, with an AUC of 0.864. This is consistent with the findings obtained in this study. However, the validity of the model is challenged by its dependence on adjustable parameters by the operator.

Apart from predicting NSLN status based on imaging features, such as ultrasound or clinical features, the role of molecular markers has also been explored. Metalloprotease-1 (41) and cytokeratin 19 mRNA copy (42, 43) have been suggested to be highly correlated with NSNL metastasis. Prediction models based on molecular markers usually showed a high specificity and sensitivity, but the time taken for intraoperative measurements and the high cost may be the reasons why they are difficult to extend.

Our nomogram provides an individual prediction of the probability of having negative NSLNs for patients with positive SLNs. A woman with 2 positive SLNs, out of 5 SLNs harvested, the preoperative US showed the presence of lymph node hilum and pathology revealed tumor LVI, might be considered to have a 20% risk of having negative NSLNs, which implies that the patient is at high risk of additional nodes metastasis and ALND should be recommended clinically. However, the study result is limited and requires much more validation before it can be applied to clinical reasoning.

Machine learning has been applied to different tumors as an innovative method for cancer prediction and prognosis (15, 16). It provides excellent accuracy through a continuous ML approach. According to Ayer et al. (17), a prediction model that enrolled 48,774 patients yielded an AUC of 0.965 and could distinguish malignant from benign mammographic findings. Such precision is unfathomable in alternative models. Typically, an AUC above 0.7 is regarded as reasonable. Madekivi et al. (19) narrowed down to seven variables by utilizing XG-Boost's capabilities for self-learning and eventually developed a nomogram with an AUC of 0.80. This study compared the performance of three ML methods, and the best classification model was selected. To reduce the number of variables, LASSO conducted 500 times iterations and selected the features with more than 300 repeated occurrences. This showed a well predictive effectiveness in the validation cohort.

The inclusion of US-related variables and the application of the ML approach are the two aspects of innovation in our prediction model. Eventually, the four variables included in the development of the nomogram were the number of positive SLNs, total number of SLNs harvested, LVI, and lymph node hilum status. The AUC values of 0.759 and 0.705 were used for the training and validation cohorts, respectively. Therefore, in comparison with other previous studies, the validation cohort in this study had a higher AUC value for the fewer variables. The continuous predictors were categorized using restricted cubic splines, which are better suited for the daily practice of clinicians. However, some limitations of the study were noted. First, the number of patients enrolled in this study is far from satisfaction. The inclusion criteria entailed only the patient who received both SLNB and ALND and this dramatically reduced the number. The nomogram lacked external validation due to the limitation in numbers, which meant that patients had to be studied as a single center although we included patients in multicenter. This study was short of a large sample that is technically required for ML to support a more convincing result. On the other hand, our pathology department was failed to accurately depict the size of SLN metastases at the beginning, owing to the inclusion of patients over a long time interval. This was significantly regretted because the existing literature already supports a relationship between SLN metastasis size and NSLN status.

Overall, the trend of de-escalation of axillary surgery is inevitable. However, the selection of appropriate patients remains a crucial issue to be addressed. It is essential to have some multicenter, prospective trials, such as Z0011, to upgrade the guidelines. This study revealed more areas that need future research to validate our findings.

## Data Availability Statement

The raw data supporting the conclusions of this article will be made available by the authors, without undue reservation.

## Author Contributions

QW collected the data and was a major contributor to writing the manuscript. LD analyzed and interpreted the patient data. YJ made substantial contributions to the conception. HZ designed the work and revised it. All authors contributed to the article and approved the submitted version.

## Conflict of Interest

The authors declare that the research was conducted in the absence of any commercial or financial relationships that could be construed as a potential conflict of interest.

## Publisher's Note

All claims expressed in this article are solely those of the authors and do not necessarily represent those of their affiliated organizations, or those of the publisher, the editors and the reviewers. Any product that may be evaluated in this article, or claim that may be made by its manufacturer, is not guaranteed or endorsed by the publisher.

## Supplementary Material

Supplementary Figure S1 The line graph shows the relationship between the continuous variables and the risk of non-sentinel lymph nodes (NSLNs) metastasis status by using restricted cubic splines analysis. Y-axis means risk ratio and the X-axis means values of variables. **(A)** age; **(B)** tumor color Doppler flow imaging (CDFI); **(C)** total number of SLNs harvested; **(D)** number of positive SLNs; **(E)** longitudinal/transverse axis ratio of tumor; **(F)** longitudinal/transverse axis ratio of lymph nodes; **(G)** proportion of positive SLNs; **(H)** transverse axis of tumor; **(I)** transverse axis of lymph nodes.

Supplementary Table S1 Differences in clinicopathological characteristics between the training and validation sets.

Supplementary Table S2 Comparisons of the predictive performance of validation sets among the three algorithms models.
