# Supplementary figures and images for: Application of the Machine-Learning Model to Improve Prediction of Non-Sentinel Lymph Node Metastasis Status Among Breast Cancer Patients
Source: Front Surg. 2022 Apr 25;9:797377. doi: 10.3389/fsurg.2022.797377 (PMC9082647; doi:10.3389/fsurg.2022.797377)

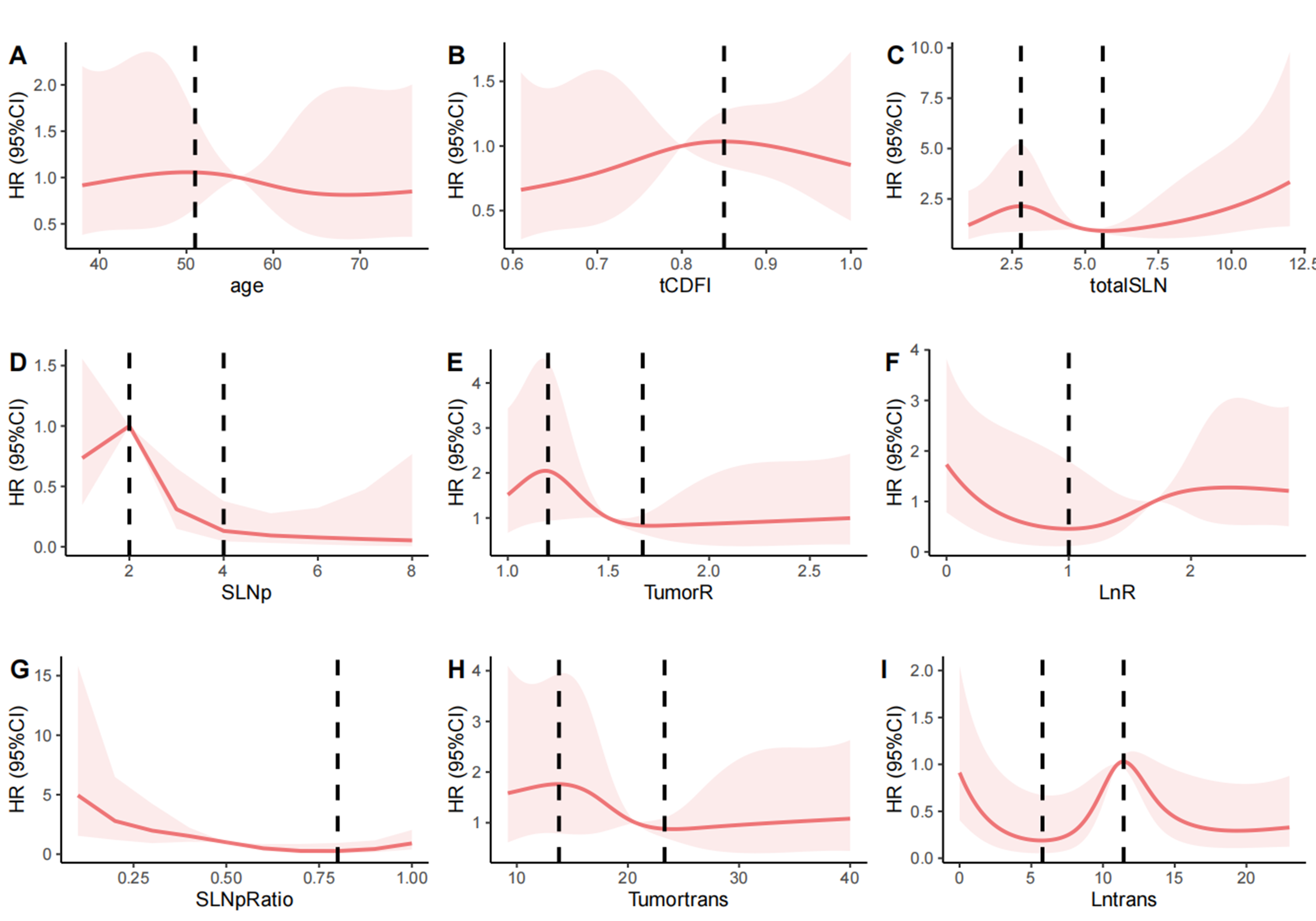

Supplement: Supplementary Figure S1 — The line graph shows the relationship between the continuous variables and the risk of non-sentinel lymph nodes (NSLNs) metastasis status by using restricted cubic splines analysis. Y-axis means risk ratio and the X-axis means values of variables. (A) age; (B) tumor color Doppler flow imaging (CDFI); (C) total number of SLNs harvested; (D) number of positive SLNs; (E) longitudinal/transverse axis ratio of tumor; (F) longitudinal/transverse axis ratio of lymph nodes; (G) proportion of positive SLNs; (H) transverse axis of tumor; (I) transverse axis of lymph nodes. [file Image_1.tif]
