# Supplementary material for: Application of the Machine-Learning Model to Improve Prediction of Non-Sentinel Lymph Node Metastasis Status Among Breast Cancer Patients
Source: Front Surg. 2022 Apr 25;9:797377. doi: 10.3389/fsurg.2022.797377 (PMC9082647; doi:10.3389/fsurg.2022.797377)
Supplement: Supplementary Table S1 — Differences in clinicopathological characteristics between the training and validation sets. [file Data_Sheet_1.docx]

Table S1: Differences of clinicopathological characteristics between the training and validation sets.

|  | Training set (n=157) | Validation set (n=58) | Entire set (n=215) | Pvalue |
| --- | --- | --- | --- | --- |
| Age |  |  |  |  |
| ＜51 | 58 (36.9%) | 22 (37.9%) | 80 (37.2%) |  |
| ≥51 | 99 (63.1%) | 36 (62.1%) | 135 (62.8%) | 1 |
| Tumor location |  |  |  |  |
| Non-UOQ | 74 (47.1%) | 31 (53.4%) | 105 (48.8%) |  |
| UOQ | 83 (52.9%) | 27 (46.6%) | 110 (51.2%) | 0.504 |
| NSLNs metastasis |  |  |  |  |
| Yes | 71 (45.2%) | 25 (43.1%) | 96 (44.7%) |  |
| No | 86 (54.8%) | 33 (56.9%) | 119 (55.3%) | 0.902 |
| Ultrasonic features |  |  |  |  |
| Transverse diameter of tumor (mm) |  |  |  |  |
| ≤14 | 27 (17.2%) | 12 (20.7%) | 39 (18.1%) |  |
| 15~22 | 66 (42.0%) | 25 (43.1%) | 91 (42.3%) |  |
| ≥23 | 64 (42.0%) | 21 (43.1%) | 85 (42.3%) | 0.772 |
| Transverse/longitudinal axis ratio of tumor |  |  |  |  |
| ≤1.2 | 45 (28.7%) | 15 (25.9%) | 60 (27.9%) |  |
| 1.3~1.7 | 65 (41.4%) | 26 (44.8%) | 91 (42.3%) |  |
| ≥1.8 | 47 (41.4%) | 17 (44.8%) | 64 (42.3%) | 0.886 |
| Transverse diameter of lymph nodes (mm) |  |  |  |  |
| ≤6 | 35 (22.3%) | 16 (27.6%) | 51 (23.7%) |  |
| 7~11 | 52 (33.1%) | 17 (29.3%) | 69 (32.1%) |  |
| ≥12 | 70 (33.1%) | 25 (29.3%) | 95 (32.1%) | 0.7 |
| Transverse/longitudinal axis ratio of lymph nodes |  |  |  |  |
| ＜0.94 | 36 (22.9%) | 15 (25.9%) | 51 (23.7%) |  |
| ≥0.94 | 121 (77.1%) | 43 (74.1%) | 164 (76.3%) | 0.789 |
| Tumor margin |  |  |  |  |
| Irregular | 10 (6.4%) | 5 (8.6%) | 15 (7.0%) |  |
| Regular | 147 (93.6%) | 53 (91.4%) | 200 (93.0%) | 0.784 |
| Tumor CDFI |  |  |  |  |
| ＜0.85 | 98 (62.4%) | 37 (63.8%) | 135 (62.8%) |  |
| ≥0.85 | 59 (37.6%) | 21 (36.2%) | 80 (37.2%) | 0.979 |
| Lymphatic echogenicity |  |  |  |  |
| none | 35 (22.3%) | 14 (24.1%) | 49 (22.8%) |  |
| low | 76 (48.4%) | 32 (55.2%) | 108 (50.2%) |  |
| others | 46 (29.3%) | 12 (20.7%) | 58 (27.0%) | 0.446 |
| Absence of lymph node hilum |  |  |  |  |
| No or not described | 145 (92.4%) | 50 (86.2%) | 195 (90.7%) |  |
| Yes | 12 (7.6%) | 8 (13.8%) | 20 (9.3%) | 0.266 |
| Pathological features |  |  |  |  |
| Histology |  |  |  |  |
| ductal | 151 (96.2%) | 57 (98.3%) | 208 (96.7%) |  |
| lobular | 5 (3.2%) | 1 (1.7%) | 6 (2.8%) |  |
| others | 1 (3.2%) | 0 (1.7%) | 1 (2.8%) | 0.7 |
| T stage |  |  |  |  |
| I-II | 152 (96.8%) | 58 (100%) | 210 (97.7%) |  |
| III | 5 (3.2%) | 0 (0%) | 5 (2.3%) | 0.387 |
| Estrogen receptor status |  |  |  |  |
| negative | 27 (17.2%) | 10 (17.2%) | 37 (17.2%) |  |
| positive | 130 (82.8%) | 48 (82.8%) | 178 (82.8%) | 1 |
| Progesterone receptor status |  |  |  |  |
| negative | 48 (30.6%) | 15 (25.9%) | 63 (29.3%) |  |
| positive | 109 (69.4%) | 43 (74.1%) | 152 (70.7%) | 0.614 |
| Proliferation index (Ki-67) |  |  |  |  |
| ＜14% | 29 (18.5%) | 14 (24.1%) | 43 (20.0%) |  |
| ≥14% | 128 (81.5%) | 44 (75.9%) | 172 (80.0%) | 0.465 |
| Her-2 overexpression |  |  |  |  |
| negative | 116 (73.9%) | 45 (77.6%) | 161 (74.9%) |  |
| positive | 41 (26.1%) | 13 (22.4%) | 54 (25.1%) | 0.705 |
| lymphovascular invasion |  |  |  |  |
| no | 122 (77.7%) | 39 (67.2%) | 161 (74.9%) |  |
| yes | 35 (22.3%) | 19 (32.8%) | 54 (25.1%) | 0.164 |
| SBR stage |  |  |  |  |
| I-II | 87 (55.4%) | 36 (62.1%) | 123 (57.2%) |  |
| III | 70 (44.6%) | 22 (37.9%) | 92 (42.8%) | 0.471 |
| Total number of SLNs harvested |  |  |  |  |
| ≤2 | 31 (19.7%) | 17 (29.3%) | 48 (22.3%) |  |
| 3~5 | 63 (40.1%) | 20 (34.5%) | 83 (38.6%) |  |
| ≥6 | 63 (40.1%) | 21 (34.5%) | 84 (38.6%) | 0.324 |
| number of positive SLNs |  |  |  |  |
| 1~2 | 106 (67.5%) | 43 (74.1%) | 149 (69.3%) |  |
| 3~4 | 37 (23.6%) | 11 (19.0%) | 48 (22.3%) |  |
| ≥5 | 14 (8.9%) | 4 (6.9%) | 18 (8.4%) | 0.645 |
| proportion of positive SLNs |  |  |  |  |
| ＜0.8 | 120 (76.4%) | 36 (62.1%) | 156 (72.6%) |  |
| ≥0.8 | 37 (23.6%) | 22 (37.9%) | 59 (27.4%) | 0.055 |

UOQ: Upper-outer quadrant; NSLNs: non-sentinel lymph nodes; CDFI: color Doppler flow imaging; SBR grade: Scarff-Bloom-Richardson grade; SLNs: sentinel lymph nodes.

Table S2: Comparisons of the predictive performance of validation sets among the three algorithms models.

| Tuning parameters of RF model |  |  |  |
| --- | --- | --- | --- |
| mtry | Accuracy | Kappa | AccuracySD |
| **1** | **0.7** | **0.39** | **0.11** |
| 2 | 0.68 | 0.35 | 0.12 |
| 3 | 0.69 | 0.36 | 0.12 |
| 4 | 0.67 | 0.33 | 0.13 |
| 5 | 0.67 | 0.32 | 0.13 |
| 6 | 0.67 | 0.32 | 0.13 |
| 7 | 0.67 | 0.33 | 0.13 |
| 8 | 0.67 | 0.33 | 0.13 |
| 9 | 0.67 | 0.32 | 0.13 |
| 10 | 0.67 | 0.32 | 0.13 |
| Tuning parameters of SVMs model |  |  |  |
| cost | ROC | Sensitivity | Specificity |
| 0.3 | 0.7 | 0.84 | 0.52 |
| 0.5 | 0.69 | 0.84 | 0.52 |
| 0.7 | 0.68 | 0.84 | 0.52 |
| 1 | 0.66 | 0.84 | 0.52 |
| 2 | 0.69 | 0.84 | 0.52 |
| 3 | 0.66 | 0.84 | 0.52 |
| 4 | 0.68 | 0.84 | 0.52 |
| **5** | **0.72** | **0.84** | **0.52** |
| 6 | 0.68 | 0.84 | 0.52 |
| 7 | 0.68 | 0.84 | 0.52 |
| 8 | 0.68 | 0.84 | 0.52 |
| 9 | 0.68 | 0.84 | 0.52 |
| 10 | 0.69 | 0.84 | 0.52 |
| 11 | 0.68 | 0.84 | 0.52 |
| 12 | 0.67 | 0.84 | 0.52 |

Bold is the best parameter
